# Pharmacotherapy in pulmonary arterial hypertension: a systematic review and meta-analysis

**DOI:** 10.1186/1465-9921-11-12

**Published:** 2010-01-29

**Authors:** Christopher J Ryerson, Shalini Nayar, John R Swiston, Don D Sin

**Affiliations:** 1 Department of Medicine, University of British Columbia, Vancouver, Canada

## Abstract

**Background:**

Previous meta-analyses of treatments for pulmonary arterial hypertension (PAH) have not shown mortality benefit from any individual class of medication.

**Methods:**

MEDLINE, EMBASE, and the Cochrane Central Register of Controlled Trials were searched from inception through November 2009 for randomized trials that evaluated any pharmacotherapy in the treatment of PAH. Reference lists from included articles and recent review articles were also searched. Analysis included randomized placebo controlled trials of at least eight weeks duration and studies comparing intravenous medication to an unblinded control group.

**Results:**

1541 unique studies were identified and twenty-four articles with 3758 patients were included in the meta-analysis. Studies were reviewed and data extracted regarding study characteristics and outcomes. Data was pooled for three classes of medication: prostanoids, endothelin-receptor antagonists (ERAs), and phosphodiesterase type 5 (PDE5) inhibitors. Pooled relative risks (RRs) and 95% confidence intervals (CIs) were calculated for mortality, 6-minute walk distance, dyspnea scores, hemodynamic parameters, and adverse effects. Mortality in the control arms was a combined 4.2% over the mean study length of 14.9 weeks. There was significant mortality benefit with prostanoid treatment (RR 0.49, CI 0.29 to 0.82), particularly comparing intravenous agents to control (RR 0.30, CI 0.14 to 0.63). Mortality benefit was not observed for ERAs (RR 0.58, CI 0.21 to 1.60) or PDE5 inhibitors (RR 0.30, CI 0.08 to 1.08). All three classes of medication improved other clinical and hemodynamic endpoints. Adverse effects that were increased in treatment arms include jaw pain, diarrhea, peripheral edema, headache, and nausea in prostanoids; and visual disturbance, dyspepsia, flushing, headache, and limb pain in PDE5 inhibitors. No adverse events were significantly associated with ERA treatment.

**Conclusions:**

Treatment of PAH with prostanoids reduces mortality and improves multiple other clinical and hemodynamic outcomes. ERAs and PDE5 inhibitors improve clinical and hemodynamic outcomes, but have no proven effect on mortality. The long-term effects of all PAH treatment requires further study.

## Background

Pulmonary arterial hypertension (PAH) is a progressive and debilitating disease characterized by a pathological increase in the resistance of the pulmonary circulation [1,2]. The increased pulmonary vascular resistance (PVR) leads to right ventricular dysfunction, exertional impairment, and premature death [3]. The United States national prospective registry for primary pulmonary hypertension reported the median survival for the idiopathic form of PAH to be only 2.8 years without treatment [3].

There is currently no cure for PAH, however the past two decades have seen significant advances with the development and clinical implementation of a number of medications that specifically target the aberrant regulatory and structural changes in the pulmonary arterial bed [4,5]. Three classes of drugs have been developed and approved for the treatment of PAH: prostanoids, endothelin-1 receptor antagonists (ERAs), and phosphodiesterase type 5 (PDE5) inhibitors. All three classes of medication have been shown to favorably affect hemodynamic parameters as well as improve functional capacity and exercise tolerance [4]. Although all three classes of drugs have been evaluated in well-designed clinical studies, only one early trial of intravenous epoprostenol was able to detect improvement in mortality in functional class III and IV patients [6]. No other treatment has been demonstrated to have an impact on mortality. Futhermore, adequately powered trials could be considered ethically inappropriate considering the documented symptomatic and functional benefits of many treatments in PAH. This illustrates the role of a meta-analysis in determining the improvement in mortality with these other treatments.

Two meta-analyses have reviewed the treatments of PAH [7,8]. A meta-analysis by Macchia et al in 2007 included some patients with non-PAH pulmonary hypertension and the results of several trials have been reported since this publication [7]. A meta-analysis by Galiè et al published in 2009 concluded that PAH treatment improved mortality, however this conclusion is limited by the pooling of all three classes of PAH treatment and the inclusion of multiple doses of medication, some of which are not approved for clinical use due to either increased adverse effects or lack of efficacy [8]. The failure to include unpublished data in this meta-analysis may have also introduced a publication bias.

We sought to improve upon these previous meta-analyses by addressing these issues. By pooling the available literature, we sought to determine the effect of these classes of medication on total mortality and secondarily to assess their impact on other clinical endpoints, including dyspnea, exercise tolerance, hemodynamics, and adverse effects.

## Methods

### Literature search

We performed a literature search using the MEDLINE and EMBASE databases to identify randomized controlled trials that evaluate the effects of pharmacotherapy on outcomes in PAH. We used the following search terms: pulmonary hypertension, pulmonary arterial hypertension, pulmonary artery hypertension, pulmonary vascular disease, pulmonary heart disease, and pulmonary cardiac disease. The details of the search strategy are summarized in Additional file 1. We also searched the Cochrane Central Register of Controlled Trials and examined bibliographies of retrieved articles and other major review articles. Our search included articles and conference abstracts published from database inception to November 2009 (PUBMED from 1950 to November 2009, EMBASE from 1980 to November 2009). No language restriction was applied. Studies were included if they evaluated adults with PAH and had a follow-up of eight weeks or more. Studies were excluded if they were not double-blind randomized placebo-controlled trials. The only exceptions were studies that evaluated intravenous agents since the use of placebo may be considered unethical in some jurisdictions. We used the Jadad score and the Cochrane Collaboration's tool for assessing methodologic quality and risk of bias, and accepted only those trials with a score of three or greater (two or greater for trials of intravenous agents) using these scales [9,10]. We included studies published in abstract form if sufficient information was available to assess methodologic quality. The literature search, data abstraction, and methodologic grading were performed independently by two authors (CJR and SN) using a predefined standardized data abstraction form. All discrepancies were resolved by iteration and consensus.

### Endpoints

The primary end point was total mortality from any cause. Secondary end points included 6 minute walk distance (6 MWD), Borg dyspnea scores, functional class (New York Heart Association (NYHA) or World Health Organization (WHO) scores), hemodynamic parameters, and adverse events. Hemodynamic parameters included mean pulmonary artery pressure (mPAP), mean right atrial pressure (mRAP), cardiac index, and pulmonary vascular resistance (PVR) obtained by right heart catheterization. For studies reporting PVR in Woods units, we multiplied this value by 80 to obtain the PVR in dyn-sec/cm^5^.

### Statistical analysis

We pooled the data for each end point from individual studies to produce summary effect estimates. Where possible, the endpoints were analyzed based on intention-to-treat. We used the p value or CI when pooling data for studies reporting significance in multiple manners (e.g. p value, CI, standard error, standard deviation). For dichotomous outcomes we calculated a relative risk (RR) and 95% confidence interval (CI). We calculated weighted mean differences and 95% CI for continuous variables. In studies reporting only the placebo-corrected mean change, we used this value for the mean change in the intervention group and assigned a value of 0 for the placebo group. Heterogeneity was examined using a X^2 ^test. For outcomes with significant heterogeneity (p ≤ 0.10) we used a random-effects model to pool the data; otherwise, a fixed-effects model was used. All analyses were conducted using Review Manager statistical software (version 5.0.17 Cochrane Collaboration; Oxford, England). A *p*-value of less than 0.05 was considered significant.

## Results

**Figure 1:**
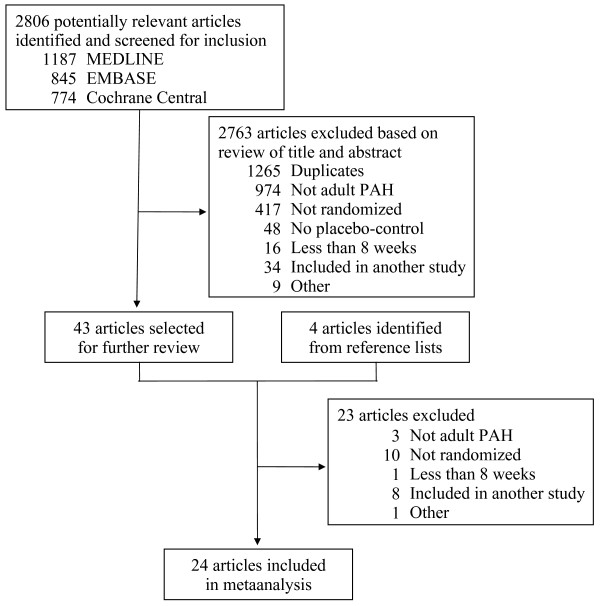
**Study selection**. PAH, pulmonary arterial hypertension.

Twenty-four studies (N = 3758 patients) satisfied the inclusion criteria (Figure 1). The characteristics of these studies are summarized in Additional file 2.

### Prostanoids

This analysis was based on 1404 patients from eleven studies that evaluated prostacyclin or prostacyclin analogues, including intravenous epoprostenol and treprostinil, subcutaneous treprostinil, inhaled iloprost and treprostinil, and oral beraprost [6,11-20]. One study included patients with non-PAH forms of pulmonary hypertension [15]. We analyzed data from this study only for the outcomes that described patients with PAH separately. All studies were double-blind randomized controlled trials excluding three studies that compared intravenous epoprostenol to conventional therapy without placebo [6,13,14]. One study of intravenous trepostinil compared trepostinil to placebo [18]. The study of intravenous treprostinil and one study of inhaled treprostinil were published in abstract form only, but provided sufficient information for analysis [17,18].

**Figure 2:**
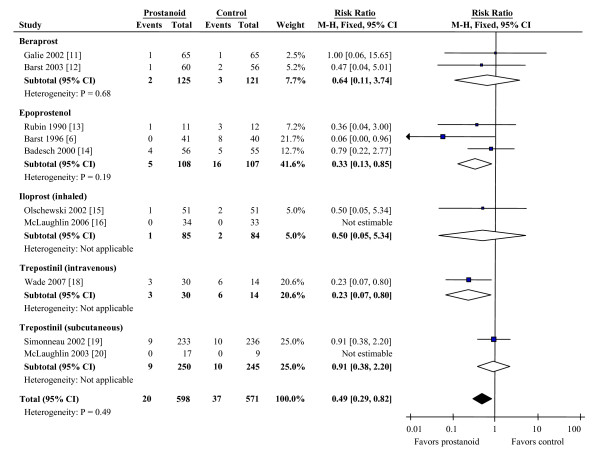
**Effects of prostanoids on mortality during treatment of PAH**. CI, confidence interval; M-H, Mantel-Haenszel method.

**Figure 3:**
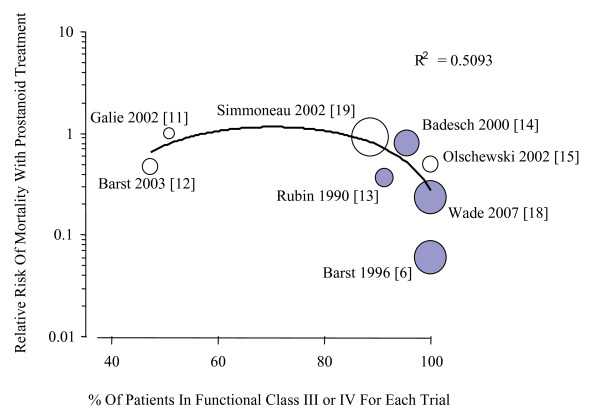
**Relationship between mortality rate and functional class severity in individual trials**. Relationship between the relative risk of mortality with prostanoid treatment compared to placebo and the proportion of patients in each trial with functional class III or IV symptoms (weighted linear regression). Studies with a greater proportion of functional class III or IV patients showed a greater reduction in mortality (R^2 ^= 0.5093). Shaded circles represent studies of intravenous prostanoids.

Mortality outcomes were available for ten studies (Figure 2). Overall, compared with conventional therapy or placebo, prostanoids reduced mortality by 51% (RR 0.49, CI 0.29 to 0.82). This benefit was maintained when using a random effects model (RR 0.54, CI 0.32 to 0.94). Reduction in mortality was more pronounced when comparing only intravenous agents versus placebo (RR 0.30, CI 0.14 to 0.63), and when correlating risk of mortality to the proportion of patients in the trial with functional class III or IV symptoms (Figure 3).

6 MWD outcome was analyzed in ten of the eleven studies. Borg dyspnea score and NYHA/WHO functional class were analyzed in seven studies, and hemodynamic changes in eight studies. Prostanoids were associated with improvements in the 6 MWD (mean placebo-corrected improvement 29.4 meters, CI 18.1 to 40.7), Borg dyspnea score (improvement -1.10, CI -1.61 to -0.59), WHO and NYHA functional class improvement (RR 3.39, CI 1.56 to 7.36), and hemodynamic parameters (Table 1). Effects were greater for all endpoints when intravenous studies were analyzed separately.

**Table 1:** Summary of Change in Hemodynamic Outcomes with Intervention Compared to Placebo/Control

Drug Class	mPAP	mRAP	PVR	Cardiac Index
Prostanoids	-4.2 (-6.2, -2.1)	-1.6 (-2.3, -0.9)	-291 (-401, -182)	0.32 (0.12, 0.52)
Endothelin receptor antagonists	-4.9 (-6.6, -3.2)	-1.4 (-2.9, 0.2)	-245 (-316, -174)	0.30 (0.09, 0.51)
Phosphodiesterase type 5 inhibitors	-4.2 (-5.7, -2.7)	-1.8 (-2.8, -0.8)	-192 (259, 126)	0.39 (0.15, 0.63)*

Data are reported as mean placebo/control-corrected change (95% confidence interval)

mPAP, mean pulmonary artery pressure; mRAP, mean right atrial pressure; PVR, pulmonary vascular resistance

* reported only in Galie et al, 2005 [29]

Adverse events were reported in six of the eleven studies [11,12,14,16,19,20]. We analyzed events that were reported in three or more individual studies. Adverse events that were significantly increased in the intervention arm are reported in Table 2. These included jaw pain, diarrhea, peripheral edema, headache, and nausea.

**Table 2:** Summary of Significant Adverse Effects with Intervention Compared to Placebo/Control

Event	Number of studies	Number of participants	RR	95% CI
Prostanoids*				
Jaw pain	5	893	4.87	2.01 to 11.76
Diarrhea	4	826	2.62	1.40 to 4.89
Peripheral edema	3	652	2.22	1.22 to 4.07
Headache	4	782	1.96	1.10 to 3.47
Nausea	5	893	1.57	1.23 to 2.00

Phosphodiesterase type 5 inhibitors*				
Visual disturbance	3	567	3.83	1.19 to 12.30
Dyspepsia	3	567	3.77	1.92 to 7.43
Flushing	3	567	2.11	1.31 to 3.39
Headache	3	567	1.73	1.19 to 2.51
Limb pain	3	567	1.67	1.10 to 2.53

CI, confidence interval; RR, relative risk

* RR & 95% CI calculated for adverse events reported in three or more studies

### Endothelin receptor antagonists

There were 1273 patients from eight studies that evaluated the use of ERAs, including oral ambrisentan, bosentan, and sitaxsentan [21-28]. Drug dosing varied between and within studies. Summary effect estimates were calculated for some doses including ambrisentan 5 mg daily, bosentan 125 mg twice daily, and sitaxsentan 100 mg daily. These doses were chosen for our meta-analysis because they were 1) the most commonly reported in the retrieved studies, 2) associated with lower incidence of adverse effects; and 3) the standard recommended doses in current practice.

**Figure 4:**
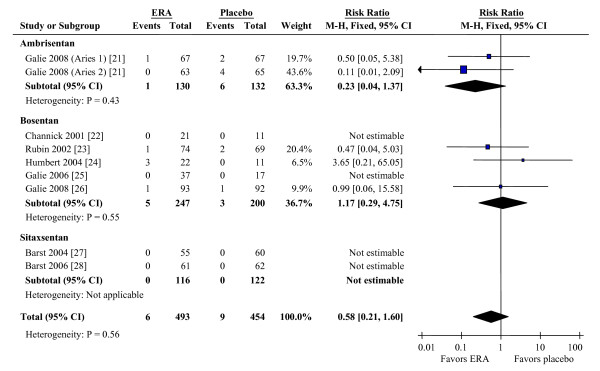
**Effects of ERAs on mortality during treatment of PAH**. CI, confidence interval; ERA, endothelin receptor antagonist; M-H, Mantel-Haenszel method.

Mortality data were available for all studies (Figure 4). Overall, compared to placebo, ERAs were not associated with a significant change in mortality (RR 0.58, CI 0.21 to 1.60). Data for 6 MWD was available for seven studies, NYHA/WHO functional class for six, and Borg and hemodynamic changes for five. Benefits were seen in 6 MWD (mean placebo-corrected improvement 38.0 m, CI 27.2 to 48.7), Borg dyspnea score (improvement -0.57, CI -0.99 to -0.15), functional class improvement (RR 1.67, CI 1.23 to 2.29), and most hemodynamic parameters (Table 1). The two trials of ambrisentan did not report hemodynamic outcomes; however, the effect size for 6 MWD and Borg dyspnea scores was greater in the ambrisentan groups than in either the bosentan or sitaxsentan groups.

Adverse events were reported in all studies, but not all adverse events were reported in each study. We analyzed events that were reported in three or more individual studies. There were no adverse effects that were significantly increased in the intervention arm. Abnormal liver function tests were reported in all studies, however only five studies provided a definition of this adverse effect, with four studies using a transaminitis greater than three times the upper limit of normal, and one study using five times the upper limit of normal [21,25-28]. No effect was seen when combining all studies, however a significant increase was found when analyzing data from the studies of bosentan (RR 2.34, CI 1.05 to 5.23).

### Phosphodiesterase type 5 inhibitors

Phosphodiesterase inhibitors were assessed in three studies including a total of 950 patients [29-31]. One study included patients randomized to placebo or three doses of sildenafil with results reported for each dose [29]. The second study titrated sildenafil dose to effect with 80% of patients receiving an 80 mg dose three times daily [30]. The tadalafil study randomized patients to placebo or four doses of tadalafil [31]. Outcome reporting was most complete for the 40 mg group which had the optimal therapeutic effect in this trial. Summary effect estimates were therefore calculated using the 80 mg groups for sildenafil and 40 mg group for tadalafil.

**Figure 5:**
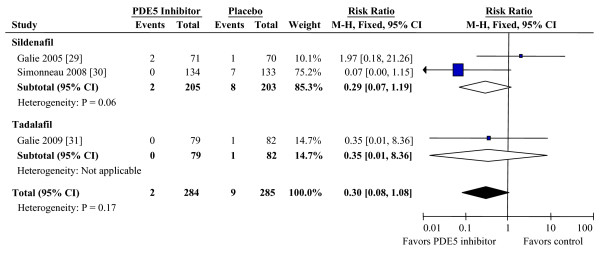
**Effects of PDE5 inhibitors on mortality during treatment of PAH**. CI, confidence interval; PDE5, phosphodiesterase type 5; M-H, Mantel-Haenszel method.

Mortality data were available for all three studies (Figure 5). Compared to placebo, PDE5 inhibition was not associated with significant change in mortality (RR 0.30, CI 0.08 to 1.08). 6 MWD was reported in three studies and hemodynamic changes in only the two sildenafil sudies. Benefits were seen in 6 MWD (mean placebo-corrected improvement 33.7 m, CI 22.5 to 44.8) and all reported hemodynamic parameters (Table 1). Borg dyspnea score and WHO/NYHA FC were reported in only one study and were therefore not analyzed.

We analyzed adverse events that were reported in all three studies. Adverse effects that were significantly increased are reported in Table 2. These included visual disturbance, dyspepsia, flushing, headache, and limb pain.

### Other treatments

Two additional studies satisfied inclusion criteria, but were not analyzed. Terbogrel, a thromboxane receptor antagonist, was evaluated in one trial [32]. This trial was terminated prematurely after the recruitment of seventy-one patients due to excessive leg pain and the subsequent high rate of non-compliance in the intervention group. Based on an intention-to-treat analysis, terbogrel improved pharmacologic endpoints, but had no significant impact on 6 MWD or hemodynamics. Rosuvastatin was assessed for six months in one trial of sixty patients [33]. 6 MWD was a secondary outcome with no change found following rosuvastatin treatment. One German paper examined the effects of molsidomine on gas exchange and hemodynamics in primary pulmonary hypertension, but did not provide enough information for analysis [34].

## Discussion

The present study demonstrates that treatment with the prostacyclin and prostacyclin analogue class significantly improves mortality in patients with PAH. Prostanoids, ERAs, and PDE5 inhibitors provide symptomatic relief and improve the functional as well as hemodynamic status of patients with PAH.

The overall prognosis of PAH is very poor with an untreated median survival as short as 3.6 years [3]. In the present study, the overall mortality in the untreated arm was 4.2% over 14.9 weeks. Thus, mortality reduction is a major goal of pharmacotherapy. However, owing to relatively small sample sizes and short duration of follow-up, the effect of various pharmacotherapies on mortality has been controversial. A recent meta-analysis by Galiè et al concluded that PAH treatment improved mortality, however this conclusion was limited by the combination of all three classes of PAH treatment [8]. Additionally, this meta-analysis included multiple doses of medications, some of which are not approved for clinical use due to lack of efficacy or increased adverse effects. The relatively narrow search strategy used in this prior meta-analysis did not identify three additional studies that were included in the present study, including two abstracts and one full-text publication [17,18,20]. We therefore build upon this meta-analysis by separately analyzing individual classes of PAH treatment, by including data only for approved medication doses, and by expanding the search to capture studies published in abstract form. In addition, we include a fourth study that has been recently published [31].

The present study indicates that mortality reduction can be achieved using prostacyclin and prostacyclin analogues. Based on the pooled estimate, the number needed to treat to prevent one death would be thirty-two patients treated for sixteen weeks. This mortality signal is driven primarily by studies of intravenous prostanoids, particularly those studies that included a greater percentage of patients with functional class III or IV symptoms. With intravenous epoprostenol or treprostinil, only eight patients would require treatment for twelve weeks to prevent one death. The studies of intravenous therapy were among the first trials performed in PAH and typically included patients with the most severe disease. The placebo arm in these trials had a high mortality rate of 18%, improving the ability to detect mortality benefit from treatment.

Non-intravenous prostanoids, ERAs and PDE5 inhibitors were not associated with a change in mortality. However, these studies often excluded patients with the most severe disease or allowed concurrent therapy with other pulmonary vasodilators. These factors may have lowered the mortality rate in these studies and thus limited the ability to demonstrate improved mortality with these treatments. Treatment with ERAs resulted in a 42% non-significant improvement in mortality in the 947 subjects included in the pooled analysis. Considering the baseline mortality of only 2% in the placebo group of these studies, approximately 7,000 subjects would be required to detect this degree of mortality improvement with ERA treatment. This calculation illustrates that such a benefit of ERAs will not be shown with additional studies of similar design. In contrast, based on the relative mortality in the treatment and control groups, the pooled analysis of PDE5 inhibitors is only slightly underpowered to detect a significant difference in mortality.

The proven mortality benefit of intravenous prostanoids is consistent with the present guidelines, which recommend the use of intravenous epoprostenol as first-line therapy for patients with poor functional class (e.g. NYHA Class IV patients). Despite the lack of proven mortality benefit, non-intravenous prostanoids, ERAs, and PDE5 inhibitors provide improvements in functional class, exercise tolerance, and pulmonary hemodynamics. These drugs may therefore be reasonable therapies for patients with mild to moderate disease with significant functional limitations.

The benefits of these drugs must be carefully balanced against possible toxicities. Bosentan is associated with an increased risk of transaminitis, however our meta-analysis found no evidence of this risk with other ERAs such as sitaxsentan and ambrisentan. These data are consistent with previous studies. Two doses of sitaxsentan have been studied as an alternative in patients who failed bosentan therapy due to transaminitis, with only one of twelve patients developing a non-fatal and reversible transaminitis after thirteen weeks of sitaxsentan therapy [35]. This effect appears to be dose-related with the standard dose of 100 mg daily being associated with fewer episodes of liver toxicity [27]. In a second study, only one of thirty-six patients discontinuing bosentan or sitaxsentan developed a transient transaminitis upon starting ambrisentan [36]. The data from the current study, while unable to conclude an absence of liver toxicity with sitaxsentan and ambrisentan, do provide further evidence that these ERAs have less liver toxicity than bosentan.

There are several limitations to this study. First, most of the included trials had a relatively small sample size and short follow-up. Thus, the effect of these drugs on long-term mortality and duration of survival improvement is uncertain, particularly for the ERA and PDE5 inhibitor classes. Second, pooling all trials within each class of medication can be criticized since trials were still heterogeneous, even within a single class. The relatively few trials for any single intervention also limited the ability to perform analyses on individual drugs within each class. Third, while several results of this meta-analysis are positive, it is not entirely clear that outcomes such as a small change in mPAP or small increases in 6 MWD (less than 30 meters) have a strong clinical impact. Fourth, we did not directly evaluate the impact of combined therapies, making it unclear whether an individual agent or the combination itself provides a more beneficial outcome. Only four randomized controlled studies have directly examined the potential benefits of combination versus single agent therapy [16,24,30,37]. The primary endpoint was not met in three of these studies [16,24,37]. The single study showing benefit in the primary endpoint reported a small, but statistically significant placebo-corrected improvement of 28.8 m in 6 MWD [30]. Finally, current guidelines recommend several other treatments for PAH [4]. Supplemental oxygen and diuretics are recommended for symptomatic control while warfarin and calcium channel blockers are recommended in some forms of PAH. Our search did not identify any randomized placebo-controlled trials that evaluated oxygen, diuretics, warfarin, or calcium channel blockers, though several observational studies suggest their benefit in PAH [38-40].

## Conclusions

The present robust meta-analysis suggests that prostanoids, ERAs, and PDE5 inhibitors all confer a therapeutic benefit. Of these, only intravenous prostacyclins has a proven survival benefit, particularly in patients with severe disease. Non-intravenous prostanoids, ERAs, and PDE5 inhibitors have not been shown to improve mortality, however these agents have not been adequately studied in patients with the most severe disease. Additional studies will be required to determine the optimal dose and duration of these therapies in exacting the best possible outcomes at the lowest cost and risk of adverse events for patients.

## List of abbreviations

6 MWD: 6-minute walk distance; CI: 95% confidence interval; ERA: endothelin receptor antagonist; mPAP: mean pulmonary artery pressure; mRAP: mean right atrial pressure; NYHA: New York Heart Association; PAH: pulmonary arterial hypertension; PDE5: phosphodiesterase type 5; PVR: pulmonary vascular resistance; RR: relative risk; WHO: World Health Organization.

## Competing interests

The authors CJR, SN, DDS have no competing interests. JRS has received honoraria from Actelion Pharmaceuticals and Pfizer/Encysive for speaking engagements as well as participation in advisory boards for GSK, Pfizer, and Actelion Pharmaceuticals. Assistance for participation in educational activities has also been received from Actelion Pharmaceuticals and Pfizer/Encysive. JRS does not have ongoing contractual or financial relationships with any of these companies. There was no funding provided for this study.

## Authors' contributions

CJR and DDS conceived the study design. CJR and SN performed the search and data abstraction. All authors participated in data analysis and interpretation. All authors participated in drafting the manuscript. All authors read and approved the final manuscript.

## Supplementary Material

Additional file 1 **Search Filters**. Search filters used for PUBMED, EMBASE, and the Cochrane Central Register of Controlled Trials. Click here for file

Additional file 2 **Characteristics of Included Trials**. BID, twice daily; CHD, congenital heart disease; CTD, connective tissue disease; IPAH, idiopathic pulmonary arterial hypertension; mPAP, mean pulmonary artery pressure; NR, not reported; QID, four times daily; Scl, Scleroderma; TID, three times daily. * Mean dose. ^† ^Median dose. ^‡ ^Included some patients with chronic thromboembolic pulmonary hypertension; analyzed patients with pulmonary arterial hypertension that were reported separately. ^§ ^Included 61 open-label bosentan patients that were not included in this data. Click here for file
